# *Leptospira interrogans* Enolase Is Secreted Extracellularly and Interacts with Plasminogen 

**DOI:** 10.1371/journal.pone.0078150

**Published:** 2013-10-18

**Authors:** Sarah Veloso Nogueira, Brian T. Backstedt, Alexis A. Smith, Jin-Hong Qin, Elsio A. Wunder, Albert Ko, Utpal Pal

**Affiliations:** 1 Department of Veterinary Medicine, University of Maryland, and Virginia-Maryland Regional College of Veterinary Medicine, College Park, Maryland, United States of America; 2 Department of Epidemiology of Microbial Diseases, Yale University School of Public Health, New Haven, Connecticut, United States of America; The University of Texas at San Antonio, United States of America

## Abstract

*Leptospira interrogans* is the agent for leptospirosis, an important zoonosis in humans and animals across the globe. Surface proteins of invading pathogens, such as *L. interrogans*, are thought to be responsible for successful microbial persistence *in vivo* via interaction with specific host components. In particular, a number of invasive infectious agents exploit host proteolytic pathways, such as one involving plasminogen (Pg), which aid in efficient pathogen dissemination within the host. Here we show that *L. interrogans* serovar Lai binds host Pg and that the leptospiral gene product LA1951, annotated as enolase, is involved in this interaction. Interestingly, unlike in related pathogenic Spirochetes, such as *Borrelia burgdorferi*, LA1951 is not readily detectable in the *L. interrogans* outer membrane. We show that the antigen is indeed secreted extracellularly; however, it can reassociate with the pathogen surface, where it displays Pg-binding and measurable enzymatic activity. Hamsters infected with *L. interrogans* also develop readily detectable antibody responses against enolase. Taken together, our results suggest that the *L. interrogans* enolase has evolved to play a role in pathogen interaction with host molecules, which may contribute to the pathogenesis of leptospirosis.

## Introduction

 Leptospirosis is a systemic disease of humans and domestic animals. In fact, it is regarded as one of the most widespread zoonotic illnesses caused by pathogenic spirochetes of the genus *Leptospira* [1-5]. While these organisms are extremely motile, they are slow-growing obligate aerobes with an optimal *in vitro* growth temperature of 30°C and can be distinguished from other spirochetes on the basis of their unique hook-shaped ends [2]. *Leptospira interrogans* constitutes the major pathogenic leptospiral species that is responsible for human infection. *L. interrogans* can readily penetrate abraded skin and mucous membrane barriers to establish a systemic infection via haematogenous dissemination and subsequently colonizes multiple organs, particularly the kidneys and liver. While wild rodents serve as natural reservoirs, humans and a few other domesticated animals are accidental hosts in the transmission cycle of leptospirosis [3,6]. As *L. interrogans* are shed in the urine of reservoir hosts and can survive in the environment, such as in water or soil for weeks to months, proper sanitation is a key intervention in reducing the transmission of leptospirosis [7,8]. Moreover, the disease has emerged as a global health threat in impoverished populations, particularly in developing countries and tropical regions where inadequate sanitation has produced the perfect conditions for this rodent-borne disease [2]. The incidence of human infection is generally higher in the tropics than in temperate regions, but transmission to humans can occur in both industrialized and developing countries [9]. Over the past decade, a number of factors, including unexpected outbreaks during sporting events, adventure tourism, and natural disasters, have underscored the ability of leptospirosis to become a public health problem even in nontraditional settings [4]. Incidence is thought to be significantly underestimated because of the lack of awareness as well as relatively imprecise diagnosis [9]. Due to a wide diversity of clinical symptoms and manifestations shared with many other diseases, diagnosis of leptospirosis is particularly challenging and depends on a variety of laboratory assays [10]. Spirochetes can be detected in cultures of infected urine or tissue samples, and diagnostics usually employ methods based on direct detection of spirochetes or their antigens using dark-field microscopy, immunostaining, or PCR, as well as indirect approaches based on host immune responses [1,9-11].

 Although antibiotics are effective in treating leptospirosis in humans, preventive strategies such as vaccination remain an important focus of leptospirosis research due to the high case fatality rate (4-40%) [8] and lack of efficient diagnostic tools, which have in turn hampered timely initiation of treatment. In particular, efforts have focused on the identification of immunogenic and novel virulence factors [2,6,12-19] and development of subunit vaccines. Specifically, research is focused on identifying surface-associated proteins that are conserved among pathogenic isolates and serve as antigenic targets for bactericidal immune responses [2]. Cell surfaceomes, especially outer membrane (OM) proteins of pathogenic spirochetes are the focus of relatively intensive investigation [14,15,20-24]. Several candidate OM proteins have been evaluated, however, with a limited degree of success. For example, immunization with LipL32, a lipoprotein constituting more than 50% of the total OM protein content [25] and that plays a dispensable role supporting acute or chronic infection with *L. interrogans* [26], has yielded equivocal results of host protection against *Leptospira* [1,2,14,27]. 

 Human-to-human transmission of *L. interrogans* is nonexistent [1]; rather, leptospirosis is acquired from an animal source or from contaminated water or soil. Thus, transmission of leptospirosis requires continuous enzootic circulation of the pathogen among animal reservoirs and long-term persistence within the host [2]. However, the intricate mechanisms by which spirochetes evade immune defenses to persist in the host and cause disease are poorly understood. The plasmin(ogen) (Pg) system is one of the most common host defense mechanisms, constituting the central pathway for dissolution of fibrin clots [28]. This system acts as a host surveillance mechanism that is essential in maintaining tissue homeostasis and facilitates cell migration by assisting the cellular penetration of protein barriers [29]. Pg is the proenzyme of the broad-spectrum serine protease plasmin, the primary fibrinolytic enzyme that is highly abundant in human tissues and plasma. Conversion of Pg to active plasmin is mediated by proteolytic activation through a number of mammalian plasminogen activators (PA), such as tissue-type plasminogen activator (tPA) and urokinase (uPA). Plasmin is involved in intravascular fibrinolysis and degradation of extracellular matrix (ECM) materials, which is relevant for cell invasion [30]. Pg contains kringle domains, which mediate its attachment to cell surfaces by binding proteins with accessible carboxyl-terminal or internal lysine residues. Together, these data indicated that the Pg system displays a unique role in host defense and maintenance of cellular homeostasis [29].

 Certain cellular proteins integral to the glycolytic pathway, such as enolase, although primarily function as metabolic enzymes, are also known to translocate to the cell surface, where they play an important role in host-pathogen interactions [31]. In many bacterial pathogens, enolase has been found to play a major role in microbial recruitment of Pg [32]. By serving as a surface receptor for Pg, enolase could mediate microbial virulence [33,34]. Although *L. interrogans* serovar Copenhageni [19,23,35] and serovar Pomona [36] have already been shown to express multiple Pg-binding proteins, the existence of additional, more widely-known Pg-binding proteins, such as enolase, remains a possibility. Here we show that *L. interrogans* serovar Lai indeed binds human Pg via enolase that is secreted extracellularly. We also show that leptospiral enolase retains weak but measurable enzymatic activity integral to the glycolytic pathway. Identification of cell-surface proteins that are involved in host-pathogen interaction is central to our understanding of microbial pathogenesis and could contribute to the development of novel preventive strategies against infection.

## Materials and Methods

### Ethics Statement

The hamster serum samples used in the current work originated from Yale University from a study approved by the Institutional Animal Care and Use Committee (Yale University IACUC Protocol #2011-11424). The serum samples were obtained through cardiac puncture after euthanasia of the hamsters using CO_2_, and animals were handled according to the above-mentioned Yale University IACUC approved protocol.

### Bacteria

 A human pathogenic strain, *L. interrogans* serovar Lai str. 56601 [37], was used in this study. The bacteria were grown at 30°C in liquid Elinghausen-McCullough-Johnson-Harris (EMJH) media.

### Production of recombinant enolase and antibody

 The *L. interrogans* open reading frame (LA1951) encoding enolase was amplified by PCR using specific primers: LA1951 sense: 5’-CGG AAT TCC TCT CAT CAC TCT CAA ATT CA-3’ and LA1951 antisense: 5’-CCG CTC GAG TAA ATT ATA AAA AGT TTC CC-3’. Recombinant enolase was produced in *Escherichia coli* using the bacterial expression vector pGEX-6P-1 (GE Healthcare). Purification of the protein, including removal of the glutathione-S-transferase fusion tag, was performed as detailed by the manufacturer (GE Healthcare). Generation of murine polyclonal antibodies against recombinant enolase as well as determination of titer and specificity of the antibodies using ELISA and immunoblotting analyses were performed as detailed previously [38,39].

### Assessment *of L. interrogans* outer membrane proteins

*L. interrogans* outer membrane (OM) and protoplasmic cylinder (PC) fractions were isolated as described previously [40] with minor modifications. Five hundred milliliters of *L. interrogans* was grown to mid to late log phase, centrifuged at 10,000 rpm for 20 min, washed with PBS, and finally resuspended in 20 mL of ice cold membrane isolation buffer [20 mM Tris-HCl, pH 9.0, 1 M NaCl, 2 mM EDTA] containing 27% sucrose. The solution was stirred with a magnetic bar at room temperature (RT) for 2 h, after which, the sucrose concentration was reduced to 13% by the addition of the same buffer and centrifuged at 7650 g for 30 min. The supernatant was collected and further centrifuged at 141,000 g for 2 h. The resulting pellet was resuspended in 6 mL buffer, layered onto a discontinuous sucrose gradient (56%, 42%, 26%), and centrifuged at 100,000 g for 16 h at 4°C. Then, the OM (upper band) and PC (lower band) were removed by needle aspiration, diluted 5 to 7 fold in cold buffer, and centrifuged at 141,000 g for 4 h at 4°C. The resulting OM pellet was resuspended in 1 mL buffer, applied to 12 mL of a continuous 10-42% (wt/wt) sucrose gradient, and centrifuged at 100,000 g for 16 h at 4°C. Finally, the OM pellet was removed by needle aspiration, diluted 5 to 7 fold in cold PBS, centrifuged at 141,000 g for 4 h at 4°C, and resuspended in 50-100 µL PBS containing 1 mM PMSF. Equivalent amounts of whole-cell lysate, OM, and PC were separated by SDS-PAGE and immunoblotted with antibodies specific for enolase, the known OM protein LipL32, and known inner membrane protein LipL31 [25].

### Identification of secreted proteins of *L. interrogans*

*L. interrogans* were grown until mid-log phase, and the viability of the spirochete culture was determined by microscopy. The supernatant samples were collected from cultures of intact viable cells by mild centrifugation and filtered through a 0.2 µm membrane. The bovine serum albumin (BSA) was removed from the collected media using a commercial kit (ProteoPrep^TM^ Blue Albumin Depletion Kit, Sigma). Protein concentration in the supernatant was quantified, and identification of specific secreted proteins was accomplished by two-dimensional (2D) SDS-PAGE and immunoblotting.

### Gel electrophoresis and immunoblotting

 Two-dimensional gel electrophoresis was performed as detailed [41] with the following modifications. Samples were solubilized in rehydration solution composed of 7 mM urea, 2 M thiourea, 0.5% (v/v) Triton X-100, 0.5% (v/v) IPG buffer pH 3-10, and 60 mM DTT. Immobiline DryStrips (GE Healthcare) were placed in rehydration solution overnight [41,42]. After isoelectric focusing, the strips were incubated in equilibration buffer containing 50 mM Tris-HCl pH 8.8, 6 M urea, 29.3% (v/v) glycerol, 2% (w/v) SDS, 0.002% (w/v) bromophenol blue, and 0.1% DTT and resolved using SDS-PAGE. Gels were finally stained with Coomassie Brilliant Blue or transferred to a membrane for immunoblotting using specific antibodies against recombinant enolase or a control leptospiral protein, LipL31, as detailed [41]. 

 Detection of enolase-specific antibody response during host infection was accomplished by immunoblot analysis as detailed [38,39]. Briefly, recombinant enolase was separated by SDS-PAGE and transferred to a nitrocellulose membrane before immunoblotting using antisera collected from hamsters that were infected with *L. interrogans* serovar Copenhageni strain Fiocruz L1-130.

### Plasminogen binding assay

 For cellular assays, microtiter wells were coated with *L. interrogans* cells in the presence of glutaraldehyde, which facilitates immobilization of cells [31]. Briefly, 10^7^ cells were coated in 1% glutaraldehyde in phosphate buffered saline (PBS), incubated for 10 min at 37°C, and washed with PBS to remove unattached cells. Wells were then blocked with 1% BSA in PBS for 1 h and incubated with human Pg (hPg - 0.5, 1.0, and 2 µg) (Sigma) for an additional hour. Competition experiments were performed by the addition of increasing concentrations (1, 2.5, and 5 µg) of recombinant enolase for 1 h prior to the addition of a constant amount (1 µg) of hPg. Another set of competition experiments was performed by the addition of anti-enolase or LipL32 antibodies or normal mouse serum (NMS) prior to the addition of hPg. Binding was assessed by incubation with anti-Pg monoclonal antibody (R&D Systems). Plates were washed three times with 0.1% Tween 20 in PBS. Horseradish peroxidase was added to the wells and incubated for 1 h at 37°C. The absorbance was measured at *A*_450_ using a microplate reader.

 In another set of experiments, wells of microtiter plates were coated with 1 μg of recombinant enolase diluted in carbonate buffer (pH 9.6). After blocking and washing, as described above, different amounts (0.5, 1.0, and 2 µg) of hPg were added to the plates. Binding was determined by incubation with anti-Pg monoclonal antibody (R&D Systems). Alternatively, the plates were also coated with 1 μg of hPg diluted in the carbonate buffer and incubated overnight at 4 °C. Plates were then blocked with 1% (wt/vol) BSA in PBS for 1 h followed by three washes with 0.1% Tween 20 in PBS. Competition experiments were performed by the addition of increasing concentrations of the lysine analogue ε-aminocaproic acid (ε-ACA, Sigma) to *L. interrogans* or Pg-coated wells. All reactions were carried out at 37°C, after which the wells were washed three times with 1% BSA in PBS. Binding was determined by incubation with anti-enolase antibody. Following three washes, the wells were developed as described above. 

### Plasminogen activation assay

 The Pg activation assay was performed by measuring the amidolytic activity of generated plasmin as detailed [31]. The wells of microtiter plates were coated with *L. interrogans* fixed in the presence of 1% glutaraldehyde and incubated with 1 µg hPg, 3 µg of a plasmin chromogenic substrate (D-valyl-L-lysyl-*p*-nitroaniline hydrochloride) (Sigma), and 15 ng of tissue plasminogen activator (tPA) (Sigma). Control experiments were performed by measuring the generation of plasmin in either the absence of tPA or presence of ε-ACA. Plates were incubated at RT for 2 h, and optical densities were read at *A*_450_.

### Degradation of fibrin in jellified matrices

 The fibrinolysis assay was performed as described [38,43] with minor modifications. Briefly, 10^7^ *L. interrogans* cells were preincubated with Pg (50 µg) for 3 h in the presence or absence of tPA (50 ng) in a final volume of 1 ml. Thereafter, the mixtures were washed three times with PBS to remove free Pg molecules. The resulting cell pellets were placed in wells of a fibrin substrate gel matrix that contained 1.25% low-melting-temperature agarose, hPg (100 µg), fibrinogen (4 mg), and thrombin in a final volume of 2 ml. Controls consisted of untreated cells (without Pg) or no cells. The jellified matrix was incubated in a humidified chamber at 37°C for 8 h. Plasmin activity was detected by the observation of clear hydrolysis haloes within the opaque jellified-fibrin-containing matrix and recorded by a Canon Rebel T2 digital camera.

### Detection of enolase on the microbial surface and its interaction with outer membrane proteins

 For detection of enolase on the surface of intact *L. interrogans*, microtiter plates were coated with intact or lysed *L. interrogans* (10^9^/ml). After blocking nonspecific sites with BSA, duplicate wells were separately incubated with antibodies against recombinant versions of enolase, LipL31, or LipL32. Bound antibody was detected using HRP-labeled secondary antibodies and TMB substrate for color development. For assessment of direct enolase interaction with OM proteins, *L. interrogans* were fractionated into OM vesicle (OMV) and PC fractions and solubilized, and protein preparations were bound to microtiter plates by overnight incubation at 4°C in PBS. After blocking in 1% BSA, fixed or increasing concentrations of recombinant enolase were added to the wells. After 1 hour of incubation at RT, the wells were washed in PBS with 0.05% Tween 20 followed by incubation with primary (anti-enolase) and secondary detection antibodies and developed with TMB substrate.

### Enzymatic assays

 Enolase activity was determined by measuring the transformation of NADH۰H^+^ to NAD^+^, as described elsewhere [38,44] with the following minor modifications. Briefly, the enzymatic reactions were performed at 25°C in 100 mM HEPES buffer pH 7.4 containing 500 mM MgSO_4_ with 2 M KCl, 56 mM 2-phosphoglycerate solution (2-PGE) (Sigma), 7 mM β-NADH (Sigma), 20 mM ADP (Sigma), lactate dehydrogenase/pyruvate kinase (PK/LDH Enzyme Solution, Sigma), and 1.6 µg of the protein in a final reaction volume of 200 µl. Enolase activity was measured in terms of the rate of reduction in the absorbance at 340 nm (i.e. increase in the production of NAD from NADH). For kinetic analyses, reactions were performed in 100 mM HEPES buffer pH 7.0, 10 mM MgSO_4_, and 7.7 mM KCl and using varied concentrations (1 to 6 mM) of 2-PGE in a final volume of 200 µl. Changes in the absorbance per minute were measured at 340 nm at 2 min intervals for a period of 20 min.

### Surface enolase activity of intact *L. interrogans* cells

 The enolase activity of intact *L. interrogans* or *E. coli* (negative control) cells was measured by a direct assay as described previously [38,44]. Briefly, bacteria were centrifuged and washed three times in a reaction buffer (100 mM HEPES, pH 7.0, 10 mM MgSO_4_, 7.7 mM KCl) with or without 2-phosphoglycerate (2-PGE) in a final volume of 200 µl. Following 5 min of incubation at RT, bacteria were removed by centrifugation (10,000 rpm for 1 min), and enzymatic activity in the supernatants was measured at *A*_340_ nm for the production of phosphoenolpyruvate. 

### Statistical analysis

 Results were presented as means (±) standard error mean. Statistical comparisons were performed using Student’s *t* test. Statistical significance was accepted for P <0.05 or lower values. 

## Results

### *L. interrogans* serovar Lai binds and activates plasminogen

**Figure 1:**
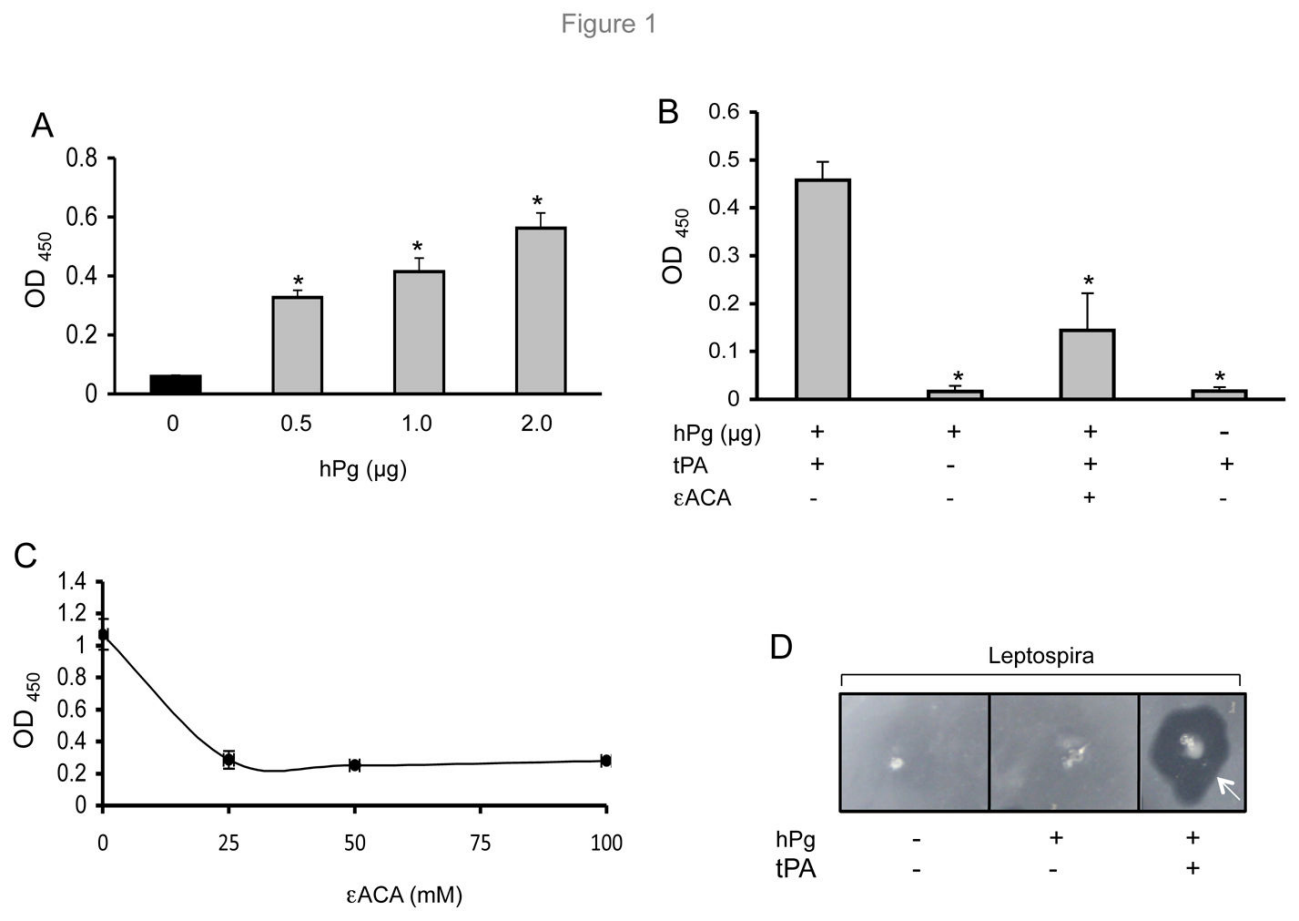
Plasminogen (Pg) binds *L. interrogans* enolase and activates plasmin. The error bars indicate the standard deviations from three independent experiments performed in triplicate. * *P* < 0.05. (A) Pg binds to *L. interrogans* in a concentration-dependent manner. Microtiter plates were coated with fixed cells and incubated with various concentrations of human plasminogen (hPg). (B) *L. interrogans* converts Pg into plasmin in the presence of tissue plasminogen (tPA). Well-bound *L. interrogans* cells were incubated with hPg (1µg) and a chromogenic substrate (D-valyl-L-lysyl-*p*-nitroaniline hydrochloride) in the presence or absence of tPA and a known Pg inhibitor, a lysine analogue, ε-ACA. (C) A Pg inhibitor blocks *L. interrogans*-hPg interaction. Microtiter plates were coated with fixed *L. interrogans* and increasing concentrations (0 to 100 mM) of ε-ACA were incubated with a fixed amount (1µg) of hPg. (D) Fibrinolytic activity of Pg-bound *L. interrogans*. Panels represent *L. interrogans* cells in the presence or absence of hPg and tPA. Arrow denotes fibrinolytic activity of spirochetes only in the presence of both hPg and tPA.

 The interaction between *L. interrogans* serovar Lai and human plasminogen (hPg) was investigated in order to identify a potential Pg receptor. To accomplish this, intact *L. interrogans* cells were fixed onto microtiter wells, incubated with increasing concentrations of hPg, and bound proteins were detected using secondary antibodies. The results show that hPg binds *L. interrogans* in a concentration-dependent fashion (Figure 1A). Such *L. interrogans*-hPg interaction also leads to the activation of plasmin, as in the presence of tissue plasminogen activator (tPA), spirochetes promoted the degradation of a chromogenic substrate (Figure 1B) that was blocked with the addition of a known Pg inhibitor, a lysine analogue (ε-ACA). Similarly, ε-ACA also reduced *L. interrogans*-hPg interaction (Figure 1C), suggesting an involvement of lysine residues of well-known Pg receptors, such as enolase. Additionally, as fibrinogen is one of the major plasmin substrates *in vivo*, and jellified matrices containing fibrinogen have been used to assess plasmin activity [43,45], we next evaluated whether the association of *L. interrogans* with Pg and tPA promotes fibrinolysis. As shown in Figure 1D, spirochetes readily promoted the lysis of fibrinogen, which was not detectable in the absence of either hPg or tPA. Taken together, the above series of assays indicated a specific interaction between hPg and *L. interrogans* cells, which leads to the activation of plasmin and subsequent degradation of fibrinogen.

### Enolase Specifically Interacts with Plasminogen

**Figure 2:**
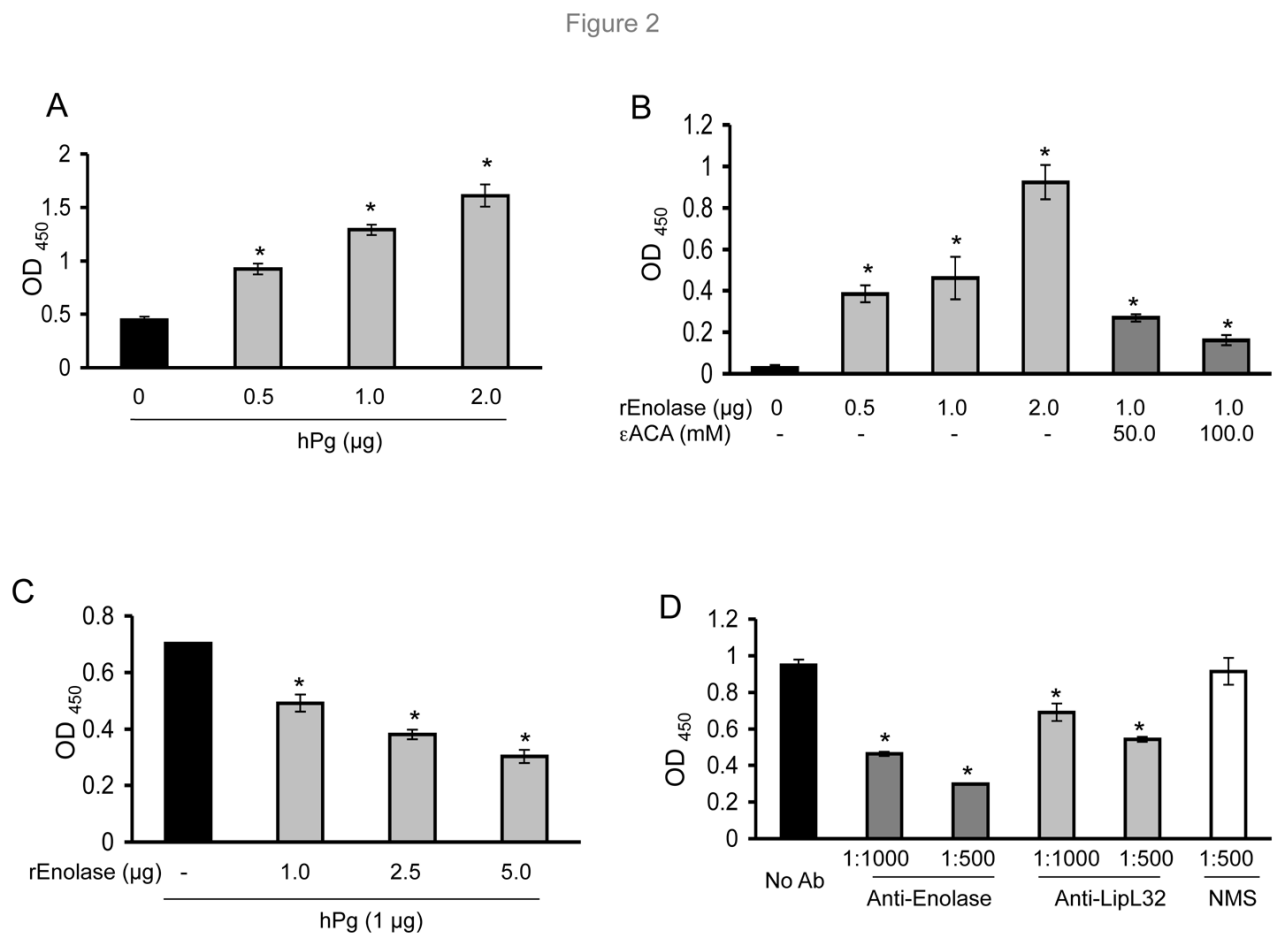
Interaction between human Pg and *L. interrogans* enolase. The error bars indicate the standard deviations from three independent experiments performed in triplicates, * P <0.05. (A) Pg directly binds to immobilized recombinant enolase. Various concentrations of hPg were incubated with a fixed amount (1 μg) of enolase immobilized on microtiter wells, and detected using Pg antibodies. (B) Recombinant enolase directly binds to immobilized Pg. Various concentrations of enolase were incubated with a fixed amount (1 μg) of hPg immobilized on microtiter wells in the absence or presence of 50 or 100 mM ε-ACA. (C) Recombinant enolase competitively inhibits binding of *L. interrogans* to Pg. Microtiter plates were coated with fixed *L. interrogans* and incubated with increasing amounts of enolase. (D) Enolase antibody significantly inhibits *L. interrogans* binding to Pg. Microtiter plates were coated with fixed *L. interrogans* and incubated in the absence or presence of anti-enolase or LipL32 antibodies prior to the addition of hPg (1µg/well). Normal mouse serum (NMS) was used as a control.

*L. interrogans* gene product LA1951 is annotated as enolase. As our previous experiments (Figure 1B and C) suggested that enolase, a well-known Pg receptor found on the surface of other pathogenic bacteria, is potentially involved in *L. interrogans*-hPg interaction, we next assessed the ability of recombinant enolase to bind hPg directly. A dose-dependent increase in the amount of bound hPg was observed when increasing amounts of hPg were added to immobilized recombinant enolase (Figure 2A). Inversely, enolase also bound to immobilized hPg and such interaction was significantly inhibited by a known competitor, a lysine analogue ε-ACA (Figure 2B), suggesting that the exposed lysine residue(s) in enolase are likely responsible for its interaction with hPg. Soluble recombinant enolase (Figure 2C) as well as polyclonal enolase antibodies (Figure 2D) both competitively reduced the interaction between hPg and intact *L. interrogans* cells. Similarly, antibodies against an abundant OM protein, LipL32, which has also been reported to have Pg-binding ability [19], were able to reduce *Leptospira*-hPg interaction. Notably, compared to with LipL32 antibodies, the magnitude of inhibition of hPg-*L. interrogans* interaction was greater in the case of anti-enolase antibodies (Figure 2D), suggesting enolase may be a more predominant Pg receptor in *L. interrogans*.

### Enolase is secreted extracellularly by *L. interrogans*

**Figure 3:**
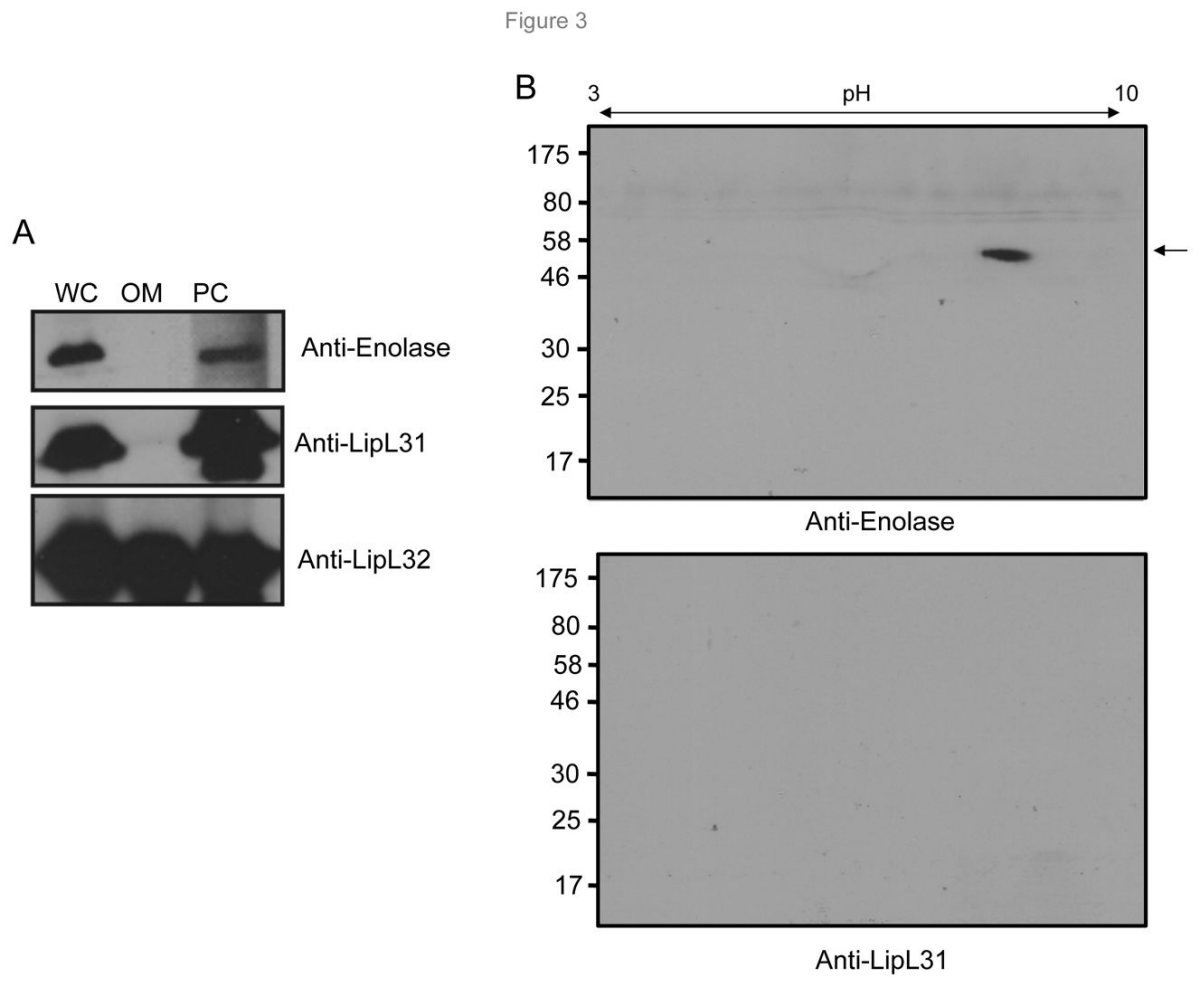
Cellular localization of *L. interrogans* enolase. (A) Subcellular localization of enolase. *L. interrogans* whole cells (WC) were separated into outer membrane (OM) and protoplasmic cylinder (PC) fractions, resolved by SDS-PAGE, and immunoblotted with antibodies specific for enolase or proteins known to localize in the OM (LipL32) or in the inner membrane (LipL31). (B) Enolase is secreted extracellularly. Viability of the *L. interrogans* culture was determined by microscopy, and the supernatant was collected from a culture of intact viable cells. The samples were filtered, concentrated, and analyzed by 2D gel electrophoresis followed by immunoblotting assays using antibodies against enolase (upper panel) or a subcellular protein LipL31 (lower panel).

 To function as a Pg receptor, a microbial ligand must be associated with its cell surface. However, similar to surface-exposed enolases in other pathogenic microorganisms [38,46,47], leptospiral enolase also lacks an amino-terminal leader peptide. To determine the localization of enolase in *L. interrogans*, we separated OM vesicle (OMV) and PC fractions from intact cells and used them in Western blot analyses. While a known OM protein, LipL32, was readily detectable in the OM fraction, enolase or an inner membrane protein, LipL31, remained undetectable in the OM fraction (Figure 3A). However, unlike LipL31, enolase was strongly detected in immunoblots of supernatants isolated from intact viable *L. interrogans* culture (Figure 3B), suggesting that enolase is secreted extracellularly.

### Detection of enolase on the microbial surface and its interaction with outer membrane proteins

**Figure 4:**
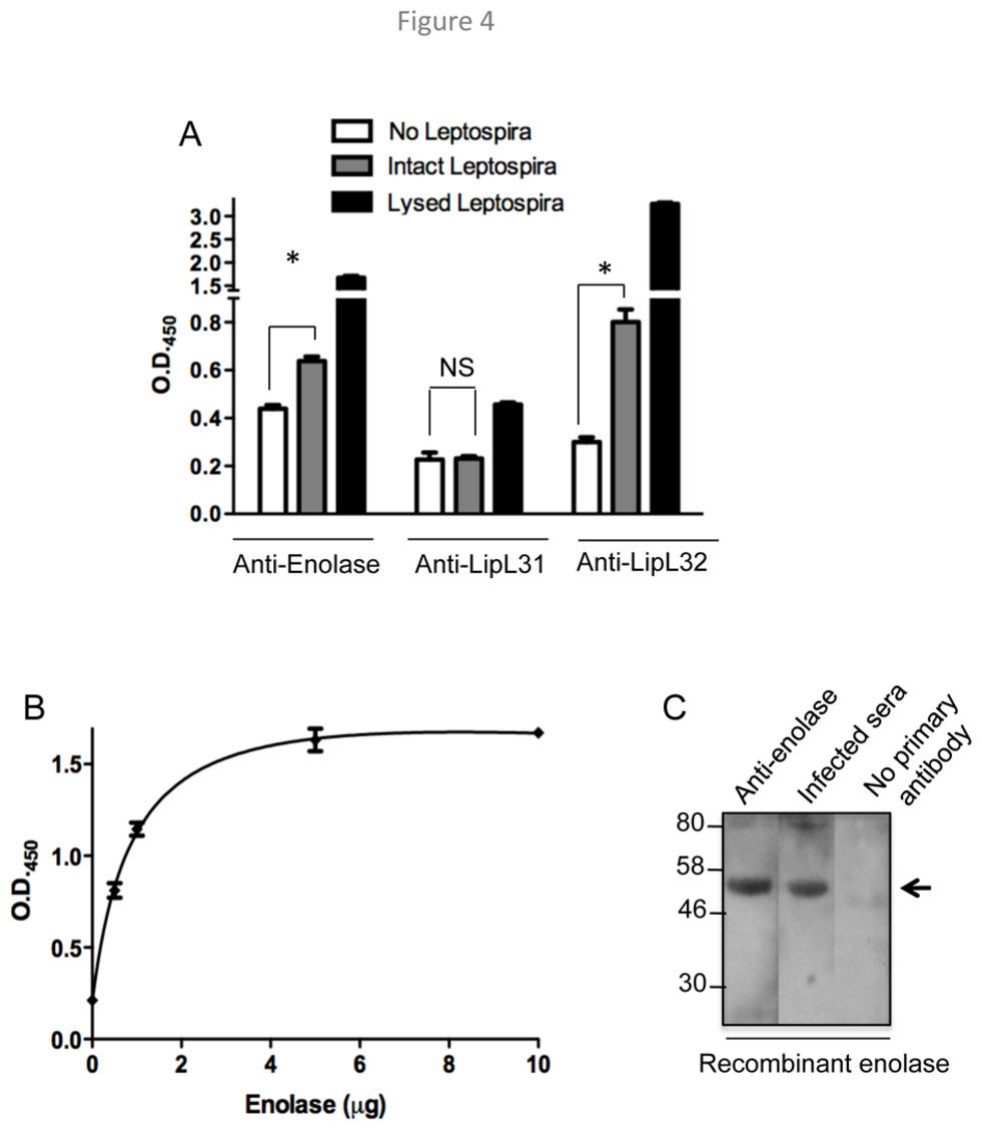
Enolase can be detected on *L. interrogans* surfaceand specifically interacts with outer membrane proteins. (**A**) Detection of enolase on the surface of intact *L. interrogans*. Microtiter plates were coated in the absence or presence of intact or lysed *L. interrogans* (10^9^/ml) and incubated with enolase antibody. Bound antibody was detected using HRP-labeled secondary antibodies and TMB substrate for color development, which was recorded at *A*_450_. A known surface-exposed and outer membrane protein, LipL32, and a subsurface inner membrane protein, LipL31, were used as controls. (**B**) Interaction of enolase with OM proteins. A fixed amount (1µg) of solubilized proteins from isolated OM vesicles were coated on microtiter plates and assessed for binding with increasing amount of recombinant enolase, as described in panel B. The binding of enolase to immobilized OM proteins reached saturation at 5 µg, as there is a significant increase (P < 0.001) in individual OD values between 0-5 µg, while the difference between 5 and 10 µg values is nonsignificant (P > 0.05). (**C**) Recognition of recombinant enolase by infected hamster serum as assessed by immunoblotting. Recombinant enolase was probed with antibodies produced in immunized mice or antiserum collected from hamster infected with *L. interrogans*. The arrow indicates the position of enolase. Migration of protein standards is shown to the left.

 As extracellularly secreted enolase has been shown to reassociate with cell surfaces in other infectious bacteria [46], we next assessed whether enolase also binds to the leptospiral surface by examining the ability of enolase antibodies to directly bind intact, fixed yet nonpermeabilized leptospiral cells. Enolase antibodies readily bind immobilized *L. interrogans* (Figure 4A), and the binding is enhanced in a concentration-dependent manner (data not shown), suggesting that enolase is accessible at the *L. interrogans* surface. To further assess the specificity of enolase localization, microtiter plates were coated with PBS or intact or lysed *L. interrogans* and incubated with antibodies against either enolase, a known surface protein (LipL32) or a subsurface protein (LipL31). Results indicated that unlike with LipL31, antibodies against both enolase and LipL32 are significantly bound to the surface of intact bacteria, suggesting potential localization of their respective antigens on the pathogen surface (Figure 4A). To further understand the association of enolase on the leptospiral surface, we next assessed whether enolase can interact with OM proteins. OMVs were purified from *L. interrogans*, and solubilized proteins were coated on microtiter plates. Immobilized OM proteins were probed with excess recombinant enolase, and bound proteins were detected using enolase antibodies. Enolase bound to unidentified OM protein(s) in a saturable manner (Figure 4B). 

 In agreement with the above data suggesting extracellular secretion and surface association of enolase, we also found that an antibody response against the antigen is readily detectable during experimental infection of hamsters with *L. interrogans* (Figure 4C). 

### Recombinant and native surface enolase retains enzymatic activities

**Figure 5:**
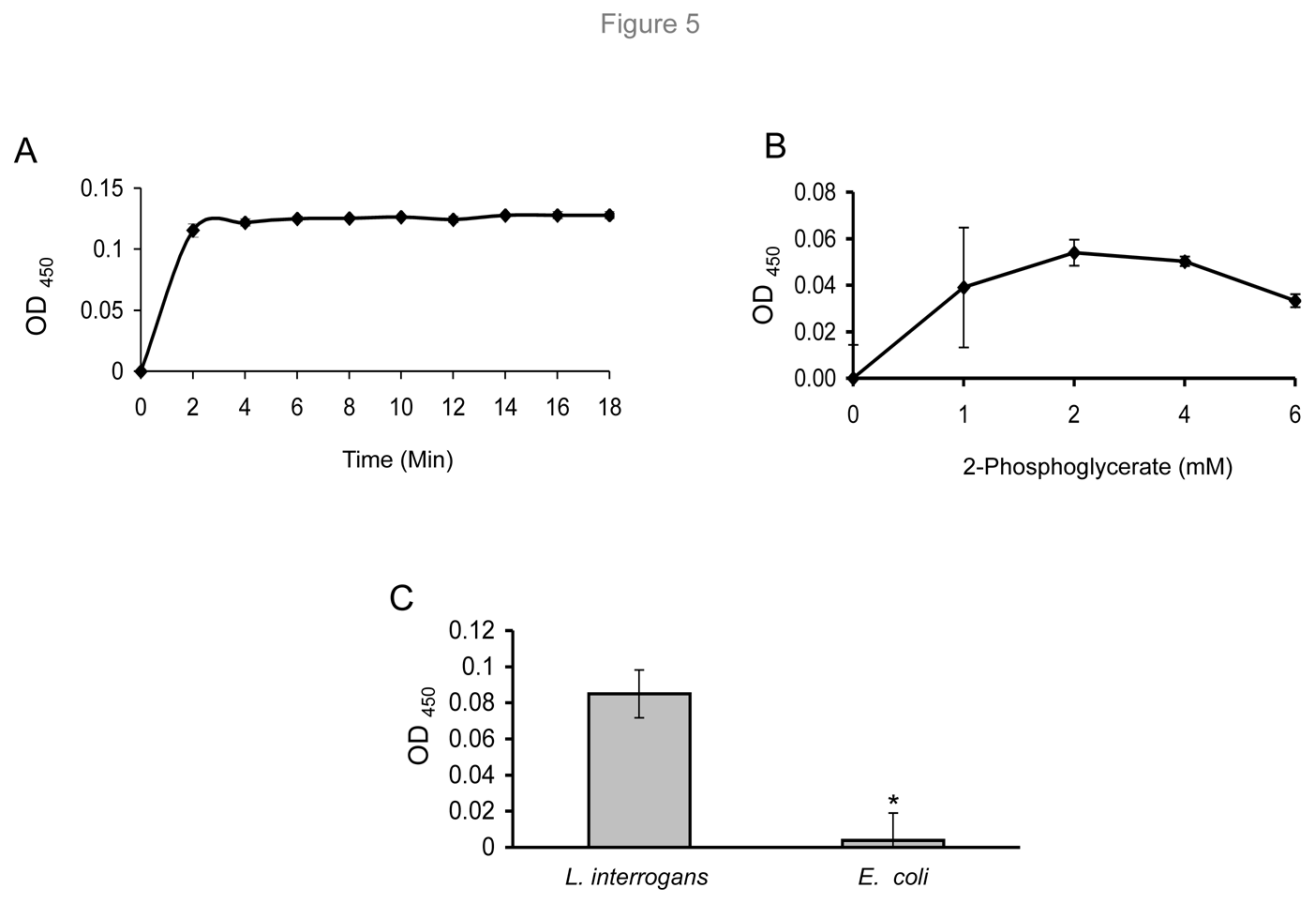
Enzymatic activities of recombinant and native surface-associated *L. interrogans* enolase. (A) Enolase activity of the recombinant enolase is highly saturable over time. Enzyme activity was measured by recoding the catalysis of 2- phosphoglycerate to phosphoenolpyruvate for a period of 20 min using 1.6 µg of recombinant enolase. (B) Substrate-dependent saturation of enzymatic activities of recombinant enolase. Increasing concentrations of the substrate (2- phosphoglycerate) were incubated with a fixed amount (4 µg) of enolase. (C) Enolase activity is detectable on the surface of intact *L. interrogans*. Conversion of 2- phosphoglycerate to phosphoenolpyruvate was used to measure enolase activity in the presence of intact *L. interrogans* or *E. coli* cells. The error bars indicate the standard deviations from three independent experiments performed in triplicates, * P <0.05.

 As enolase possesses specific Pg-binding properties and is detected on the spirochete surface, we finally assessed whether *L. interrogans* enolase retains the enzymatic activity integral to the glycolytic pathway. The activity of recombinant enolase was assessed by measuring the transformation of NADH۰H^+^ to NAD^+^ as described elsewhere [44]. The results show saturation of enolase activity over time (Figure 5A) and increasing substrate concentration (Figure 5B), suggesting measurable and specific enzymatic activities. To determine whether enolase retains enzymatic activity on the spirochete surface, conversion of 2-phosphoglycerate (2-PGE) to phosphoenolpyruvate (PEP) was measured in the presence of intact *L. interrogans* cells. The results indicated measurable enolase activity of *L. interrogans* cells but not of control Gram-negative bacteria (Figure 5C). 

## Discussion

The Pg-binding property of many pathogenic spirochetes facilitates their invasiveness, thereby supporting bacterial survival in the host [19,35,38,47-51]. Interaction of host Pg with a specific microbial surface ligand can lead to the activation of plasmin, which mediates degradation of intravascular clots and extracellular proteolysis, thus influencing a wide variety of physiological and pathological processes [9,29,30]. Here we show that *L. interrogans* enolase specifically interacts with recombinant Pg and that the native protein is secreted extracellularly by *L. interrogans*. The exact mechanism by which enolase is secreted by spirochetes remains enigmatic. As described in studies using other bacteria [46,52], enolase secretion might not be a consequence of cell lysis or membrane shedding but rather through a process in which protein structure, such as a hydrophobic α-helical domain of enolase, is a contributing factor [52]. Involvement of a secretion system also remains a possibility, as the *L. interrogans* genome encodes for type I and II secretion-like genes [2,37,53]. In either case, our data suggest that once secreted by a yet-unknown mechanism, enolase probably localizes on the bacterial surface by reassociation. Although the nature of secreted enolase binding to the *L. interrogans* surface and the identity of the cellular receptor remain interesting subjects of future investigation, a recent study involving *Streptococcus pyogenes* raises an intriguing possibility that cell surfaces play a role in enolase-Pg interaction [54]. The interaction of enolase with the cell surface is thought to produce a conformation of enolase capable of binding to host plasminogen. Despite its ability to interact with a ligand, *L. interrogans* enolase, either in recombinant form or as the native surface-associated protein, retains measurable enzymatic activities; this is an expected finding, as ClustalW analyses of enolase sequences from various microorganisms (data not shown) also show that *L. interrogans* enolase retains the motif ‘SHRSGETED’ integral to its catalytic properties [45]. Whether *L. interrogans* uses the glycolytic pathway as a source of energy [37,55] or how the enzymatic activity of enolase contributes to leptospiral physiology, however, remains unknown. Although the biological significance of enolase-Pg interaction in leptospiral virulence remains to be studied, our data showing the generation of enolase-specific antibody responses in infected hosts as well as extracellular or microbial surface-associated localization of enolase suggest that the protein may facilitate the pathogen’s infection in the host. 

Microbial-Pg interaction has been shown to assist pathogens in establishing infection in hosts [31,38,48,49,56-58]. Our initial search for potentially common virulence factors in pathogenic spirochetes infecting humans focused on enolase, which is a relatively conserved antigen among many species. Enolase has been shown to function as a Pg receptor on the cell surface of a variety of other pathogens [38,44,45,58-60]. The interactions of pathogenic spirochetes L. *interrogans*, *B. burgdorferi*, and *T. denticola* with Pg has been studied [35,38,47,49,50,61]; especially in the case of Lyme disease spirochetes, enolase-Pg interaction has been suggested to support *B. burgdorferi* survival in the vector [38]. However, potential contribution of enolase in *L. interrogans*-Pg interaction and in infectivity remains unexplored. Our current studies showing a dose-dependent inhibition of the *L. interrogans*-Pg binding activity by enolase antibodies or directly by recombinant enolase suggested that this antigen is a predominant Pg-binding protein in *L. interrogans* and also confirmed the high specificity of such interaction. The binding of Pg to its receptors is mediated by its five kringle domains, which have an affinity for lysine residues [29], and in fact, lysine-dependent binding is a salient feature of pathogen-Pg interaction [44]. Here, we show that the lysine analogue εACA and recombinant enolase significantly inhibited *L. interrogans*-Pg binding, suggesting the importance of kringle domains in such host-pathogen interaction. In addition, previous work on *S. pneumoniae* enolase revealed that Pg binding is mediated not only by C-terminal lysine residues but also by an internal Pg-binding motif with the sequence FYDKERKVY located between the amino acids 248 and 256, although full conservation of this motif is not required for optimal binding [33,62]. Notably, ClustalW analyses revealed the presence of the internal motif FYDKSKKKY located between the amino acids 251 and 259 in *L. interrogans* enolase (data not shown). Thus, we hypothesized that *L. interrogans* binds Pg via surface enolase, which facilitates conversion of bound Pg into plasmin, thereby armoring the pathogen with the potential ability to degrade fibrin and efficiently disseminate within hosts, as reported in other microorganisms [45]. 

Many microbial virulence factors are cell surface proteins that mediate pathogen interaction with specific host molecules. Accordingly, leptospiral surface proteins are likely to facilitate host cell-pathogen interaction [2] and thus contribute to virulence. Although, how enolase potentially contributes to *L. interrogans* virulence via host-Pg interaction remains a puzzling question. Leptospiral enolase lacks a recognizable leader peptide and could not readily be detectable in isolated OM preparations; however, we present evidence that the protein is secreted extracellularly. Notably, using specific antibodies, native enolase can be detected on the *L. interrogans* surface, and recombinant enolase specifically interacts with OM protein(s). These observations strongly suggest a potential reassociation of the protein with the pathogen surface. In other invasive pathogens, such as in *S. pneumoniae*, enolase is also secreted and can reassociate by interacting with receptors on the pneumococci surface [33] via Pg interaction to facilitate infection. Therefore, we speculate that *L. interrogans* enolase, either as an anchorless protein or via its potential reassociation with the microbial surface, interacts with host Pg, aiding tissue invasion by *L. interrogans*. However, why pathogenic *L. interrogans* strains, such as serovar Copenhageni, are shown to produce many additional Pg-binding proteins, such as LipL32, LIC10494, LIC12730, Lp29, Lp49, LipL40, MPL36, and LIC12238 [19,35], is perplexing. Arguably, such a large cohort of microbial ligands likely results in higher affinity of the spirochetes towards host Pg. As certain pathogenic bacteria differentially produce surface antigens in specific environments that contribute to their survival [4,63], we speculate that evolution of a diverse repertoire of Pg receptors in pathogenic *Leptospira* could be linked to the ability of this remarkable and highly invasive pathogen to infect multiple hosts or a variety of tissues within the same host, facilitating dissemination and colonization in a wide array of environments. 
